# Effects of Mediterranean Diet on Chronic Degenerative Diseases and Human Healthy Lifestyle

**DOI:** 10.3390/nu17071231

**Published:** 2025-04-01

**Authors:** Daniela Bonofiglio

**Affiliations:** 1 Department of Pharmacy, Health and Nutritional Sciences, University of Calabria, 87036 Rende, Cosenza, Italy; daniela.bonofiglio@unical.it; Tel.: +39-0984-496208; 2 Centro Sanitario, University of Calabria, 87036 Rende, Cosenza, Italy

## 1. Introduction

Noncommunicable diseases (NCDs), known as chronic diseases, including diabetes mellitus, obesity, cardiovascular diseases, chronic kidney disease, neurodegenerative diseases, and cancers, represent an important public concern, leading to prolonged disability and death. Indeed, the 2030 Agenda for Sustainable Development recognizes NCDs as a major challenge for sustainable development [1]. A crucial strategy to control NCDs is to reduce the associated main modifiable factors like unhealthy diets, physical inactivity, smoking habits, and alcohol consumption. Conversely, a healthy dietary pattern associated with correct daily lifestyle habits has been linked to a better quality of life and a reduced risk of developing NCDs [2]. Among healthy diets, the Mediterranean Diet (MD) represents one of the best dietary patterns, which links healthy food choices to a healthy lifestyle. For these reasons, the United Nations Educational, Scientific, and Cultural Organization (UNESCO) recognized the MD as an Intangible Cultural Heritage of Humanity in 2010, highlighting “a set of skills, knowledge, practices, and traditions ranging from the landscape to the table, including the crops, harvesting, fishing, conservation, processing, preparation and, particularly, consumption of food” [3]. It is based on the consumption of plant-based foods, including fruits and vegetables, whole grains, legumes, nuts, and extra virgin olive oil, which are enriched in bioactive compounds that exert beneficial effects in humans. For instance, carotenoids are pigments responsible for the yellow, orange, and red color of fruits and vegetables being a potent source of antioxidants in the diet [4]; fibers and phytosterols present in whole grains, legumes, nuts, vegetables, fruits, and extra virgin olive oil showed antioxidant, anti-inflammatory, antineoplastic activities and antiatherogenic properties [5]. Oleocanthal, a polyphenol found in extra virgin olive oil, inhibited the cyclooxygenases 1 and 2 activity [6]; resveratrol, procyanidins, and monophenols present in red wine exert antioxidant properties [7]; omega-3 polyunsaturated fatty acids and their derivatives found in fish and nuts exhibit immunomodulatory and anti-inflammatory effects, reducing the levels of pro-inflammatory cytokines [8,9,10,11,12,13]. As a graphic representation, the new MD pyramid establishes dietary daily, weekly, and occasional guidelines to follow a healthy and balanced diet, while the incorporation of cultural and lifestyle elements is one of the innovations of the pyramid. Among these elements, the importance of the regular practice of moderate-intensity physical activity is emphasized as a basic complement to the MD for balancing energy intake, maintaining a healthy body weight and for many other health benefits. Compelling evidence suggests that increasing adherence to the MD pattern is associated with a reduction in total and cause-specific mortality, and in a lower incidence of major NCDs [14,15]. In addition, recent investigations have shown that the combined effects of an MD-like pattern with regular physical activity (PA) reduced overall mortality risk [16] and all-cause mortality [17,18]. Therefore, the adoption of healthy lifestyles, which include the MD and PA, represents a priority strategy to counteract the burden of chronic diseases and promote healthier life.

The Special Issue entitled “Effects of Mediterranean Diet on Chronic Degenerative Diseases and Human Healthy Lifestyle” is composed of four original research papers and one review of literature, aiming to investigate the impact of multiple pathways specifically activated and stimulated by compounds from MD as well as by PA on human health.

Firstly, Calcaterra et al. conducted a literature review investigating the benefits of the MD in mitigating aging-related inflammation associated with childhood obesity. Analyzing 130 papers, the authors have described the crosstalk between obesity, chronic inflammation, and comorbidities in children [19]. When free fatty acids cannot be stored in the adipose tissue because the storage capacity in this compartment is exceeded, as it occurs in childhood obesity, they acquire an ectopic location determining the release of hepcidin by the liver, which induces inflammation [20,21]. The activation of the inflammatory cascade leads to the production of nuclear factor κB (NFκB) and several cytokines, including interleukin (IL)-6, IL-1, and Tumor Necrosis Factor (TNF)-α, leading to a low-grade chronic inflammatory status. It has been reported that greater adherence to the MD reduced the levels of IL-1, IL-2, IL-6, and TNF-α as well as C-reactive protein (CRP) and leptin in adolescents [22,23], and IL-17 levels in children [24]. Moreover, it has been demonstrated that adhering to the MD reduced the risk of overweight, obesity, and abdominal obesity by the age of 8. Interestingly, an inverse correlation was observed between MD adherence and different anthropometric and serum biochemical parameters, including BMI [25], waist circumference [26], insulin resistance, and high lipid levels [27]. These results have also recently been confirmed by Yurtdaş et al., who demonstrated an improvement of several metabolic parameters in obese adolescents following the MD for 12 weeks [28]. Besides the positive effects of the MD, the adherence to this dietary pattern was low among children and adolescents living in Mediterranean countries [29,30]. Thus, future studies are needed to promote the benefits of this dietary pattern and to deeply explore the specific impact of the MD on inflammaging in childhood obesity [19].

In contrast, Faienza et al. found unhealthy habits associated with a transition to the Western diet in a population of obese children and adolescents [31]. The authors have detected 12 subjects of 41 total participants having metabolic dysfunction-associated steatotic liver disease (MASLD), along with higher subcutaneous and visceral adipose tissue. Moreover, they have demonstrated that fructose intake and saturated fats were associated with MASLD in pre-school and adolescents with obesity, highlighting the need of nutritional educational programs aimed at increasing awareness among young people about the importance of a healthy lifestyle.

Findings from the study conducted by Caparello et al. also underlined the importance of promoting healthy eating habits among adolescents. Indeed, they observed a medium adherence to the MD among adolescents from Southern Italy [32]. Interestingly, the authors measured the skin carotenoid levels of participants by the spectroscopy-based device Veggie Meter^®^, which resulted in positively associated adherence to the MD evaluated by the Mediterranean Diet Quality Index (KIDMED) and MD Pyramid tests. As previously reported by other authors, Caparello et al. have demonstrated that the measurement of the skin carotenoid levels by the Veggie Meter^®^ is an objective biomarker of fruit and vegetable intake. Moreover, they have found that the skin carotenoid levels are negatively associated with BMI, highlighting the role of a healthy diet in preventing obesity [32].

In another study, Di Renzo et al. aimed to assess the differences in MD adherence in relation to age and sex, and to evaluate the impact of the adherence to the MD on body composition, according to sex [33]. The authors demonstrated that greater adherence to the MD is associated with a decrease in body weight and BMI, while no gender-related differences in the degree of MD adherence were observed in the population studied [32,34]. Of note, Di Renzo et al. have investigated for the first time the gender-related impact of the MD adherence in an Italian population in an 8-week nutritional intervention study. They found a significantly higher percentage of women consuming vegetables, nuts, and white meat than men, whereas a significantly lower percentage of women preferred fruits, legumes, fish, seafood, and “sofrito” than men. Moreover, they reported a significant increase of 19.1% and 11.1% in the Mediterranean diet adherence screener (MEDAS) scores after 8 weeks of MD treatment in men and women, respectively. Although the increase in the MD adherence was lower in women than men, a significantly higher body weight loss was reported in women than in men [33].

Finally, Bovenzi et al. have investigated the impact of MD adherence on sleep disturbances and migraine chronification in a cohort of adults from the Lazio region in Italy. Using standardized tools such as the “PREvención con DIeta MEDiterránea” (PREDIMED) score, the Pittsburgh Sleep Quality Index, and clinical scales (Headache Impact Test-6 and Migraine Disability Assessment Scale) to assess the adherence to the MD, the quality of sleep and the grade of the migraine, respectively, the authors have demonstrated that lower adherence to the MD is associated with higher migraine disability in patients with high-frequency episodic migraines and chronic migraines. Moreover, the sleep–wake disturbances were correlated with greater migraine disability [34].

Overall, all the studies included in this Special Issue provide an update on MD adherence and PA in the population in relation to age and sex and its association with different NCDs, highlighting the importance of adopting the MD model for a better quality of life. However, more comprehensive and robust evidence is needed to demonstrate that the combination of healthy diet and PA can provide the greatest health benefits.
